# Spared ulnar nerve injury results in increased layer III–VI excitability in the pig somatosensory cortex

**DOI:** 10.1038/s41684-024-01440-0

**Published:** 2024-09-30

**Authors:** Suzan Meijs, Andrew J. Hayward, Thomas Gomes Nørgaard Dos Santos Nielsen, Carsten Reidies Bjarkam, Winnie Jensen

**Affiliations:** 1 Center for Neuroplasticity and Pain, Department of Health Science and Technology, Aalborg University, Aalborg, Denmark; 2 Department of Clinical Medicine, Aalborg University, Aalborg, Denmark; 3 Department of Neurosurgery, Aalborg University Hospital, Aalborg, Denmark

**Keywords:** Cortex, Chronic pain, Sensory processing, Chronic pain, Neurophysiology

## Abstract

This study describes cortical recordings in a large animal nerve injury model. We investigated differences in primary somatosensory cortex (S1) hyperexcitability when stimulating injured and uninjured nerves and how different cortical layers contribute to S1 hyperexcitability after spared ulnar nerve injury. We used a multielectrode array to record single-neuron activity in the S1 of ten female Danish landrace pigs. Electrical stimulation of the injured and uninjured nerve evoked brain activity up to 3 h after injury. The peak amplitude and latency of early and late peristimulus time histogram responses were extracted for statistical analysis. Histological investigations determined the layer of the cortex in which each electrode contact was placed. Nerve injury increased the early peak amplitude compared with that of the control group. This difference was significant immediately after nerve injury when the uninjured nerve was stimulated, while it was delayed for the injured nerve. The amplitude of the early peak was increased in layers III–VI after nerve injury compared with the control. In layer III, S1 excitability was also increased compared with preinjury for the early peak. Furthermore, the late peak was significantly larger in layer III than in the other layers in the intervention and control group before and after injury. Thus, the most prominent increase in excitability occurred in layer III, which is responsible for the gain modulation of cortical output through layer V. Therefore, layer III neurons seem to have an important role in altered brain excitability after nerve injury.

## Abstract (web summary)

Meijs et al. perform an electrophysiological investigation of cortical responses in a pig nerve injury model, showing the role of layer III–VI neurons in altered primary somatosensory cortex excitability after nerve injury.

## Main

Peripheral nerve injuries caused by trauma, surgery or disease (for example, diabetes) can evolve into persistent, severe and refractory neuropathic pain^1^. Approximately 7–10% of the general population suffers from chronic neuropathic pain, and this is expected to increase due to the increased incidence of diabetes and increased survival after cancer therapy^2^. Peripheral neuropathic pain involves damage to or inflammation of a peripheral nerve, which alters neuronal signaling and results in increased excitability of second-order spinal neurons^3,4^, giving rise to allodynia and hyperalgesia^5^. Furthermore, patients with neuropathic pain often show signs of dysfunction in ascending and descending control pathways^2^. These peripheral and central changes contribute to altered signaling to the brain, which may result in cortical reorganization^2,6^. Chronic pain can be difficult to treat, as even neuropathies with a clearly peripheral or central origin are influenced by a complex interplay of changes along the entire neuroaxis^7,8^.

In rodents, numerous preclinical neuropathic models exist based on various etiologies of neuropathic pain^9^. These models allow invasive investigations of mechanistic changes occurring after their induction. One such model is the spared nerve injury (SNI) model, which results in denervation in the area of the transected nerves and neuropathic pain in the area of the spared nerve^10^. This model has highlighted the contribution of noninjured neurons to the neuropathic pain pathology, including ectopic firing in injured and noninjured neurons and reinnervation of the denervated area by noninjured fibers^11^. Hyperexcitability has also been shown in second-order superficial (lamina II)^12^ and deep (lamina IV)^13^ dorsal horn spinal neurons after SNI^4^. Furthermore, SNI induces substantial brain alterations involving the descending modulatory pathways, the limbic system and the prefrontal and somatosensory cortices^4,9,14–16^.

Studies in rodents have shown that activation of the primary somatosensory cortex (S1) increased immediately after SNI^14^, together with the information flow from S1 to the anterior cingulate cortex^17^. One day after SNI, S1 activation was decreased, and after 8 days, it was at a level comparable with that of sham animals^14^. This decrease in activation is thought to be related to the lack of input to the denervated S1 area^14,17^. Although S1 activation was comparable between sham and SNI rats on day 8, a functional connection with the brainstem was only present in SNI rats, which is thought to be related to plasticity in the descending modulatory pathways^14^. With novel technologies, it has been possible to show robust hyperactivity in layer V of S1 in a mouse SNI model. This was caused by decreased activation of inhibitory interneurons in layer I and layer II/III, as well as increased inhibition of these interneurons^4,18^.

Although rodents are the most well-developed models in pain research, it remains challenging to translate pharmaceutical results in these models to the clinic^19,20^. For this reason, an increasing number of large animal pain models are being developed^21^. The pig’s peripheral and central nervous system, body size and metabolism are comparable with those in humans^22,23^. Furthermore, there is evidence that pigs with neuropathic injuries respond to pharmacological substances in a similar way as humans^24^.

This study is an electrophysiological investigation of cortical excitability after nerve injury (NI) in the pig. The gyrated pig brain is much larger than the rodent brain^22^ and allows for independent intracortical recordings from different cortical layers. Therefore, the purpose of this study is twofold: to determine whether there was more S1 hyperactivity when stimulating the uninjured compared with the injured nerve and to investigate how different brain layers contribute to S1 hyperactivity. Central sensitization at the level of the spinal cord reaches its maximum within an hour after NI^3,5,25^. We hypothesize that this would result in increased excitability in layer IV within the time frame of this study.

## Results

Histological analysis was primarily used to determine the placement of the electrode contacts in the cortex. Analysis of seven pig brains showed an S1 cortical thickness of 2.4 ± 0.5 mm; low cellular density was found in layer I and higher cellular density in the deeper cortical layers. The neurons in layer I were the smallest (5 µm), while neurons in layer V were the largest (15–20 µm). No differences were observed between NI (*n* = 4) and control (*n* = 3) animals.

**Fig. 1:**
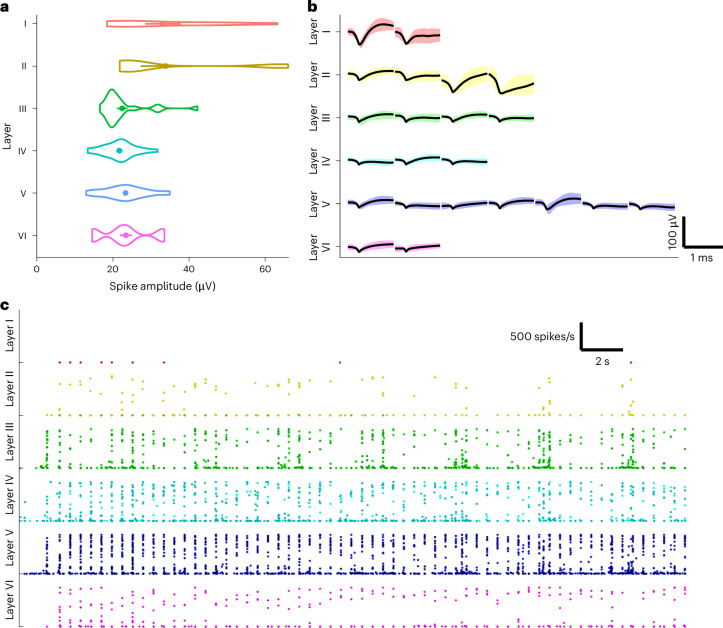
Characteristics of spikes recorded from S1 cortical layers I–VI. **a**, The average spike amplitudes in different layers of the pig cortex across all experiments. **b**, The spike waveforms recorded from different layers of the pig cortex from a representative experiment. Since layer V is relatively thick, most channels were placed in this layer, and most neurons were recorded here as well. **c**, The firing patterns in different layers of the cortex from the same experiment; different shades of the same color represent different units. The timeline starts 2 s before stimulation. Layer I had a small response to stimulation, and these channels were excluded from the analysis. Layer II was more responsive, with sometimes several spikes per stimulus. Other experiments showed more activity in layer II, yet it was never as tightly correlated with the stimulus timing as observed in layers III–VI. In layers III–VI, consistent firing can be observed after every stimulus (striped pattern). In layers III and IV, periods of higher and lower evoked activity can be distinguished at 4 s intervals. These periods of high activity also correspond to increased firing in layer II, where such bands of activity were also observed later in the experiment and in other experiments. In layers V and VI, firing patterns showed a reliable correlation to the stimulus and bands of increased activity were not observed. The length of the scale bars (in **b** and **c**) depicts the time and amplitude axes.

Spike sorting was performed to determine from how many neurons information was recorded and the characteristics of these neurons. Spikes were recorded from 1 to 3 units per channel, and most units were recorded from deeper layers, as most channels were placed in these. The evoked activity was never recorded from more than one neuron in layer I, while evoked activity was recorded from multiple units in layers II–VI (Table 1). The average spike amplitudes appeared larger in layers I and II compared with layers III–VI (Fig. 1a). Spike waveforms from a representative experiment are shown in Fig. 1b. Spontaneous firing was observed between stimulations, and corresponding firing rates were generally low (<10 spikes per second), with occasional bursts of activity displaying higher firing rates (mostly below 300 spikes per second) that often occurred in layers III and IV (Fig. 1c). Evoked firing rates consistently reached up to 740 spikes per second in every layer.

**Table 1:** The number of channels in each layer for the NI and control group

Layer	NI (*n* = 4)		Control (*n* = 3)		Total (*n* = 7)
	Channels	Neurons	Channels	Neurons	Total neurons
I	2	2	4	4	6
II	3	6	1	2	8
III	7	11	7	9	20
IV	10	13	8	12	25
V	33	38	13	20	58
VI	8	10	5	6	16
Total	63	80	38	53	133

### Brain excitability is increased after NI when the injured and uninjured nerves are stimulated

**Fig. 2:**
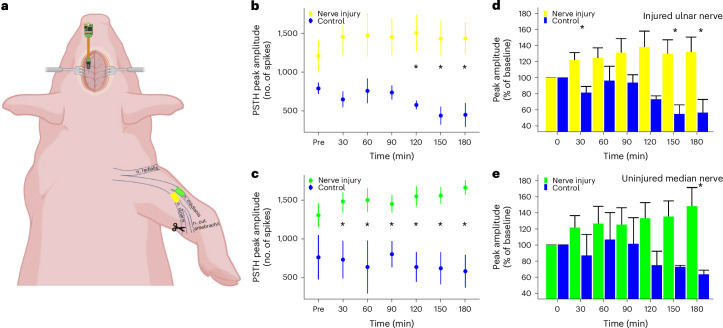
The early peak responses evoked by median and ulnar nerve stimulation are significantly increased compared with the control. **a**, Stimulation was applied to the ulnar and median nerves before and after the ulnar nerve was injured distal to the stimulation site. The cortical signals were recorded using a penetrating electrode in S1. The figure was created with BioRender.com. **b**, The peak amplitude of the early cortical response to stimulation of the ulnar nerve was significantly higher in the NI group (*n* = 6) compared with the control group (*n* = 3) from 2 h after the injury. **c**, When the median nerve was stimulated, the early cortical response was significantly higher in the NI group compared with the control group at every time point after injury of the ulnar nerve. The results are presented as estimated marginal means, and the error bars indicate the standard error of the mean. **d**,**e**, The peak amplitudes in the NI groups increase up to 140% and 150% compared with the baseline for the ulnar (**d**) and median nerves (**e**), respectively. The peak amplitude in the control group drops in the last 90 min of the experiment. *P < 0.05, RM-ANOVA. n. cut. antebrachii, antebrachial cutaneous nerves.

Spikes were binned into 1 ms bins to obtain peristimulus time histograms (PSTHs). Repeated measure analysis of variance (RM-ANOVA) was performed on the average PSTH of each pig upon stimulation of the (injured) ulnar and (uninjured) median nerve. An early and a late peak were consistently observed in the PSTHs, and the amplitude and latency of these peaks were analyzed separately. There was a significant two-way interaction between time and intervention for the early peak amplitude (*F*(6,42) = 2.942; *P* = 0.017; observed power of 0.85, RM-ANOVA). There was no significant effect of stimulating the injured or uninjured nerve (Fig. 2a). There were also no statistically significant differences between intervention and control groups at baseline (*P* = 0.12, RM-ANOVA). However, post hoc pairwise comparisons revealed that for the ulnar nerve, the early peak of the NI group (*n* = 6) was significantly greater than that of the control group (*n* = 3), only in the last three phases after NI (*P* < 0.05; confidence interval (CI) 224 to 1,737 at 180 min) (Fig. 2b), while the difference between the two groups was significant for every phase after NI (*P* < 0.05; CI 717 to 1,787 at 180 min) when the median nerve was stimulated (Fig. 2c). The same interaction was found in the statistical analysis for the normalized data shown in Fig. 2d,e (*F*(6,42) = 3.331; *P* = 0.009; observed power of 0.90, RM-ANOVA).

Residuals were normally distributed for peak latency in both time windows and for peak amplitude in the 10–19 ms time window, but not in the 21–35 ms time window. It was chosen not to study the second peak further in the RM-ANOVA analysis, and the results presented in Fig. 2 are, therefore, based solely on the early peak. There were no significant effects or interactions for peak latencies in the early time window. The mean latency (center of mass, CoM) of the first and the second peak in the median nerve was 14.9 ± 0.2 ms and 26.4 ± 0.7 ms, respectively, and in the ulnar nerve 14.6 ± 0.3 ms and 25.9 ± 0.6 ms, respectively. The latency of the early peak was consistent with the nerve fiber conduction velocity of 66 ± 8 m/s and 60 ± 8 m/s, which corresponds to Aβ fiber activation. A secondary fiber group with a conduction velocity of 26 m/s could be distinguished in some of the recordings, which corresponds to Aδ fiber activation.

### Late-evoked peak is the largest in layer III

**Fig. 3:**
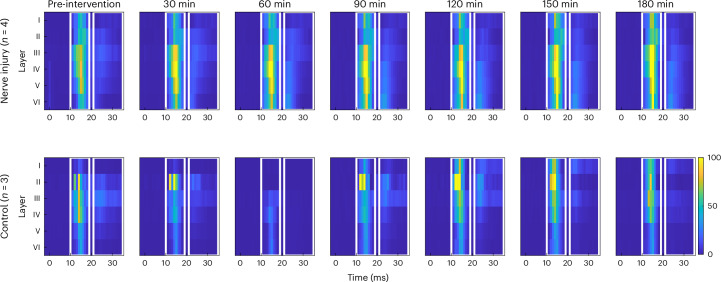
PSTHs recorded from the layers I–VI of the pig cortex. The increased excitability in deeper layers (III–VI) of the cortex after NI can be discerned (note that the number of spikes is capped at 100 spikes per bin), while superficial layers (I and II) seem to have a more constant response to electrical stimulation of the median and ulnar nerves. In the control group, the stimulus-evoked activity seems constant and concentrated in layers I–IV (apart from missing data from one animal at 60 min and possibly poor contact in layer I at the beginning of the experiments). The white boxes indicate the early and late cortical responses to stimulation. The color axis denotes the number of spikes per bin, which is capped at 100 spikes to allow visualization of the second peak.

ANOVA analysis showed that the responses evoked by stimulation of the injured and uninjured nerves were not significantly different from each other. These were, therefore, grouped in the mixed model analysis, which investigated the differences between cortical layers and the effect of SNI on each of the layers. The significant parameters of each of the models are listed in Table 2. Post hoc pairwise comparisons revealed that the amplitude of the second peak (21–35 ms after the stimulus) was significantly larger in layer III (32.1 ± 8.4 spikes/bin) compared with all other layers (*P* ≤ 0.005; CI largest difference (layer III–I) 10.5 to 28.1; CI smallest difference (layer III–IV) 5 to 16). This difference was consistent throughout the duration of the experiment but most notable in control and intervention groups before intervention (Fig. 3). Furthermore, the amplitude of the second peak was significantly larger in layer IV compared with layers I and VI, which was only significant before intervention (*P* < 0.001, mixed model pairwise comparisons; CI (layer IV–I) 0.4 to 17.4; CI (layer IV–VI) 0.9 to 12.0). For both peaks, the amplitude was significantly smaller in layer I compared with layers III–VI before intervention (*P* ≤ 0.003, mixed model pairwise comparisons; late peak layer I versus VI: *P* = 0.03, mixed model pairwise comparisons, CI −0.6 to 29.6) but not in the phases after NI and control. The amplitude of the first peak (10–19 ms after the stimulus) showed no statistically significant differences across layers. Post hoc comparisons revealed no significant differences between layers in peak latency.

**Table 2:** Significant mixed model parameters for each of the independent variables

Dependent variable	Significant factors	*F*	*P*
Early peak amplitude	Layer	*F*(5,105) = 2.751	0.022
	Intervention	*F*(1,7) = 5.704	0.048
	Layer × phase	*F*(36,199) = 2.859	<0.001
	Layer × intervention × phase	*F*(37,189) = 2.029	0.001
Late peak amplitude	Layer	*F*(5,114) = 8.152	<0.001
	Layer × phase	*F*(36,148) = 3.740	<0.001
	Layer × intervention × phase	*F*(38,94) = 3.152	<0.001
Early CoM	Phase	*F*(6,47) = 2.326	0.048
	Layer × phase	*F*(35,268) = 5.805	<0.001
	Layer × intervention × phase	*F*(38,94) = 4.314	<0.001
Late CoM	Phase	*F*(6,77) = 2.474	0.030
	Layer × phase	*F*(35,334) = 2.004	0.001
	Phase × intervention	*F*(7,22) = 2.625	0.039

### SNI results in increased evoked activity in layer III compared with the control and preinjury

**Fig. 4:**
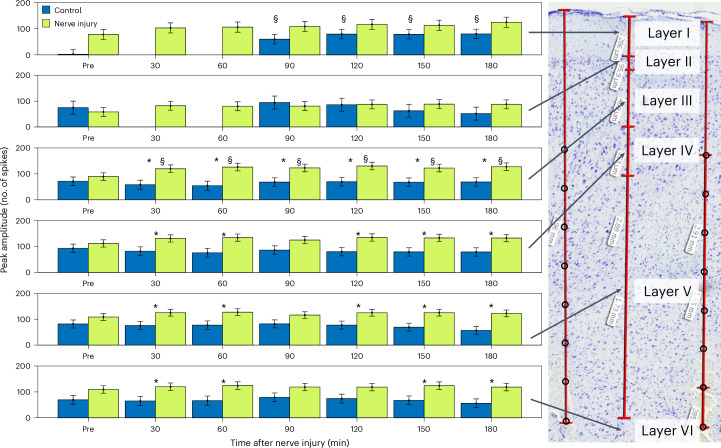
The early peak amplitude is significantly increased in layers III–VI of SI, with layer III showing the largest significant increase compared with the baseline and control. The peak amplitude of the PSTH (mean number of spikes per channel) was significantly increased in deeper layers of the cortex after NI compared with the control. In layer III, excitability was also increased after injury in the NI group compared with the baseline. In layer I, there was a lack of activity at baseline in the control group, and data are missing for this same group in the first two stimulation series after intervention. The sample sizes are the number of channels placed in each layer presented in Table 1. The results are presented as estimated marginal means, and the error bars indicate the standard error of the mean. *P < 0.05 compared with the control. ^§^*P* < 0.05 compared with the baseline, mixed model pairwise comparisons.

The mixed model pairwise comparisons only showed significant differences between the control and NI group (main effects) for the amplitude of the early peak (10–19 ms after stimulus). Figure 3 visualizes the development of both the early and late peak throughout the experiment in the NI and control group. Figure 4 shows that the early peak in layer III was significantly larger in the NI group than in the control group at all times after NI (*P* < 0.03, mixed model pairwise comparisons; CI at 60 min 23.6 to 120.3). This was also the case in layers IV and V at all times, except 90 min after NI (*P* ≤ 0.04, mixed model pairwise comparisons; CI layer IV at 60 min 11.3 to 105.9; CI layer V at 60 min 4.2 to 95.6) and in layer VI at all times, except 90 and 120 min after NI (*P* < 0.03, mixed model pairwise comparisons; CI layer VI at 60 min 9.6 to 106.5).

In line with these findings, significant main effects were found for the NI group within layer III (*F*(6,63) = 4.029, *P* = 0.002), where the amplitude of the early peak was significantly greater at all times after NI compared with before (150 min: *P* = 0.03; all other phases: *P* ≤ 0.01, mixed model pairwise comparison; CI at 60 min 8.8 to 64.7). This effect was not seen in the control group or in other cortical layers. Additionally, the peak amplitude of the early and late peaks in layer I within the control group was significantly lower before intervention compared with after (early peak: 90–180 min, late peak: 120–180 min).

## Discussion

In this study, we investigate how the injured and uninjured nerves contribute to S1 hyperactivity and how different cortical layers contribute to increased S1 excitability in the first hours after NI. The amplitude of the first peak significantly increased after NI compared with the control when the injured and uninjured nerves were stimulated. The increased amplitude was only observed in the deeper layers of the cortex (layers III–VI) and was most prominent in layer III.

### Increased excitability after NI compared with the control

A significant increase in excitability was observed in the NI group compared with the control for both the (uninjured) median and (injured) ulnar nerve. This increase is significantly different from the control group immediately after SNI for the median nerve and 120 min after SNI for the ulnar nerve. This is in line with Tøttrup et al., who found a delayed increase in the evoked S1 responses after SNI upon nonnociceptive stimulation of the injured nerve. For higher stimulation intensities, they observed a similar trend, where the greatest difference was observed between the latest recording and baseline^26^.

The (not significant) difference between the injured and uninjured nerves could be due to the partial denervation, which compromises activation of the injured fibers and thereby provides less input to the brain. Alternatively, an increase in evoked activity could be masked by a tonic increase in background activity^14^. Such sensitization of S1 was observed by Chao et al., who reported tonically increased nonevoked activity in S1 of rats in the first minutes after SNI^14^.

### Increased excitability in deeper cortical layers (III–VI)

This study showed a significantly increased cortical activation after NI compared with the control in layers III–VI but not in layers I and II. While responses for the NI group were constant in layers I and II, the contacts in layer I of the control group were unreliable, most probably due to poor tissue contact at the start of the experiment. Our results resemble the increased activity in layer V and decreased activity of inhibitory interneurons in layer I found in mice at rest 1 week and 1 month after SNI^18^. The same authors found both an increase in vasoactive intestinal polypeptide-positive cells and a decrease in somatostatin- and parvalbumin-expressing cells in layer II/III^18^. In rodents, laminar dissociation is not always clear^27^. However, here, layer II and layer III could be investigated separately due to the larger size of the pig brain and a clearer distinction between layers^28^. While no significant increase in activity was seen in layer II, the largest increase in activity was seen in layer III, which indicates distinct physiology between these layers. We speculate that the lack of change in activity in layer II may be due to the aforementioned combination of an increase in inhibition and a decrease in the activity of inhibitory interneurons. Based on the recorded firing patterns, fast-spiking interneurons dominate the results; however, methods used in this study cannot discriminate between inhibitory or excitatory function of these interneurons.

Peripheral stimulation of the median and ulnar nerve evoked two peaks of activity in the pig S1 with latencies of 15 and 26 ms. These latencies correspond to conduction velocities of Aβ (60 and 66 m/s) and Aδ (26 m/s) fiber populations. These conduction velocities are consistent with the stimulation intensity and nerve fiber activation pattern of the ulnar nerve in pigs^29^. Increased excitability was primarily found for the early peak. This increase could, therefore, be a cortical expression of allodynia, which has been found in other NI studies in pigs^24,30,31^. In rodents, it has been demonstrated that allodynia occurs in the areas innervated by the injured as well as uninjured nerves^10^, which is consistent with our findings. The second peak corresponds to Aδ fiber activation and was significantly larger in layer III compared with all other layers, both before and after intervention and in both groups. Interestingly, layers II/III are known for their gain-control function within the laminar circuitry, in particular to layer V, which projects to subcortical structures (for example, thalamus and brainstem)^32^. Layer III neurons are known to be sensitive to modulation by contextual information and arousal level^32^. Therefore, the increased late peak in this layer may be of particular significance for pain processing.

### Increased cortical excitability in layer III after NI compared with the baseline

Interestingly, we observed the greatest increase in cortical excitability after NI compared with the baseline in layer III (46% compared with 20% in layer IV), which might indicate cortical sensitization between layer IV and layer III neurons. Again, this finding points toward a specific role of layer III in pain processing. In line with previous studies^18,32^, increased activity in layer III may drive the increased excitability in layers V and VI, as layer III contains feedforward neurons projecting to layer V (ref. ^27^).

An alternative theory could be suggested based on the direct pathway proposed by Constantinople and Bruno^33^. According to the conventional indirect pathway, signals arrive at layer IV, are projected to layer III and, from there, are further conveyed to layers V and VI. Constantinople and Bruno^33^ proposed the direct pathway after the thalamus, which was found to project directly to layers V and VI, even when layer IV was inactivated by lidocaine. This manipulation removed both input to as well as signal transmission through layer IV, yet activity in layers V and VI remained almost unchanged^33^. According to the direct theory, the similar increase in excitability that was observed in layers IV, V and VI (14–20% increase after NI compared with the baseline) could be expected, if this is indeed driven by spinal hyperactivity^4,13^. The deeper cortical layers, also project back to the thalamus and brainstem, where alterations in descending modulation may occur^14^.

We found no statistically significant differences between the latencies of the responses in the different layers (*P* = 0.092 and *P* = 0.051, mixed model for the early and late peaks, respectively). This finding would be in line with the direct thalamocortical pathway theory for the deep cortical layers (layer IV–VI). However, a delay was expected for layers II/III (ref. ^33^).

### Limitations

This is one of the first studies developing a translational model of pain in pigs with recording of brain signals^21,34^. So far, there are no chronic pain studies in pigs that recorded brain signals^21^. Likewise, we started our translational work with acute studies. Therefore, we do not yet know what pain phenotype the pig will develop after the transection of the ulnar nerve. The invasiveness of the brain recordings performed in this study does not allow for the animals to survive. Nevertheless, other nerve damage models have been used in behavioral studies in the pig, including nerve crush^31^, peripheral neuritis trauma^24,31,35^ and nerve transection models^30^. These studies show that pigs develop allodynia, mechanical hyperalgesia^24,30,31^ and motor deficits depending on the injury^30,31^. Future research should combine less invasive chronic electrophysiological recordings with behavioral assessment after NI in the forelimb.

Our methodology does not provide information about the function of the neurons that we have recorded. It is, thus, difficult to determine the consequences of the hyperexcitability reported here. Immunohistochemistry, pharmacological or optogenetic methods may provide means to further investigate the functions of cortical neurons in the pig. Since the pig model is relatively novel in pain research^21^, these methods are not yet developed. It is, however, possible to stain somatostatin-, vasoactive intestinal polypeptide- and parvalbumin-expressing cells in the pig brain^36,37^, and future research should investigate in which layers of the cortex these neurons are predominant.

A nonsignificant decrease in the peak amplitudes was observed in the control group 2 h after the sham intervention. Anesthetics were kept as low as ethically acceptable to facilitate good quality evoked responses^38^. However, a buildup effect is likely to have influenced the recordings during the last 2 h of the experiment. Since this is expected to have the same effect on both groups, the difference between them indicates that NI indeed led to hyperexcitability compared with a sham intervention.

Furthermore, few channels were placed in layers I and II, which were relatively thin and layer I was sparse in neuronal density in line with previous studies^28,39^. This led to the exclusion of three irresponsive channels in layer I of one control and one intervention animal. Although we have independent recordings in these layers, this constraint makes it difficult to conclude on the results from them. The peak amplitudes were significantly smaller in layer I compared with all other layers. This is probably due to poor electrode–cell contact in layer I at the start of the experiment, as it was only found before intervention in the control group.

### Impact

Significant increased excitability in S1 layers III–VI occurred during a timescale of 3 h. These findings are in line with results from rodent SNI models and warrant longer term studies to unravel whether these changes persist and influence behavior. Changes in layers V and VI could have an influence on descending controls^14^, which has been observed in patients. This study further adds to the evidence pointing to the need to take central nervous system changes into account when developing novel treatments for peripheral neuropathic pain^7^.

## Conclusions

This study shows increased porcine cortical responses immediately after NI compared with the control when the uninjured median nerve is stimulated. This difference is also significant from 2 h after intervention, when the injured ulnar nerve is stimulated. We further show that hyperexcitability occurs in deeper cortical layers (III–VI), which could indicate an ascending mechanism. The increase in excitability was significant and most prominent compared with the baseline in layer III, which modulates excitability in cortical output layer V. Furthermore, the amplitude of the late peak was greater in layer III than all other layers, which indicates that this layer may have a significant role in pain processing.

## Methods

### Animals and study design

All experimental procedures were approved by the Animal Experiments Inspectorate under the Danish Ministry of Veterinary and Food Administration (protocol number 2016-15-0201-00884). Ten Danish Landrace pigs were included (48–52 kg, all of which were female). Female subjects were preferred due to their underrepresentation in existing literature^40,41^, despite the fact that the majority of patients with chronic pain are female^42,43^. Animals were acclimatized to the stable for 2 weeks before the experiment. Six animals underwent the NI model, and four control animals were subjected to sham intervention, as a greater heterogeneity was expected in NI compared with the control data. Technicians who were blinded to experimental groups randomly selected animals. Intervention and sham experiments were carried out interspersed so that any effect of surgical training would not influence the data in either experimental group. The cortical laminae in which the electrodes were placed were identified for seven animals (five intervention and two control). As this is one of the first studies investigating cortical changes in a porcine nerve damage model, the sample size was estimated based on typical group sizes in rodent studies of the same kind^14,17,26^ and pig studies involving NI^24,31^.

### Anesthesia

The animals were premedicated with an intramuscular injection of Zoletil Vet (1 ml per 10 kg; ketamine, 6.25 mg/ml; tiletamine, 6.25 mg/ml; zolazepam, 6.25 mg/ml; butorphanol, 1.25 mg/ml; and xylazine 6.5 mg/ml). The pigs were placed in a supine position and intubated. The jugular vein was cannulated for saline (0.9% NaCl) infusion. Anesthesia was maintained by infusion (6–10 ml/h) of propofol (10 mg/ml) and fentanyl (50 μg/ml) and ventilation with 1.5–3.0% sevoflurane. Blood pressure, heart rate, blood oxygenation, end-tidal CO_2_ and temperature were continuously monitored, and anesthetic parameters were adjusted when needed^44^. Temperature was maintained at 38 °C (±1 °C) by a forced air flow blanket placed over the animal (Mistral-Air Plus, MA1100-EU).

### Peripheral surgery

Access to the peripheral nerves in the left forelimb was achieved through an incision in the axilla and blunt dissection of the superficial pectoralis muscle. The median and ulnar nerves were separated from connective tissue before placing bipolar cuff electrodes. Additionally, a cuff electrode was implanted on each nerve branch to record nerve responses with four bipolar channels. All cuffs were insulated using additional silicone sheets and secured using ligatures. Core temperature was kept stable and local temperature was monitored using a thermocouple probe secured to a nearby muscle, as sensory nerve recruitment and conduction are dependent on temperature^45^.

A second incision was made on the lower anterior forelimb to expose the ulnar nerve. Two ligatures were loosely tied around the ulnar nerve as preparation for NI in intervention and control animals. After baseline recordings, the sutures were tied, and the nerve was cut between the sutures in the six intervention animals. The skin was closed with surgical staples during electrophysiological recordings.

### Cranial surgery

The animal was placed in a prone position, and the head was placed in a custom-built localizer box and stereotaxic frame^46^. This method allows high-precision insertion of the intracortical electrodes and prevents movement of the intracortical electrodes during other procedures. The skin was incised and retracted, and the periosteum was removed. A 5 × 5 cm^2^ square craniectomy was performed to expose the contralateral S1 region using a Dremel (8228, Dremel) with a burr drill and rongeurs. The hole extended 1 cm ipsilateral to the sagittal and posterior to the coronal suture lines (see also ref. ^34^). Bone screws were placed lateral and anterior to the square to act as ground and reference for the intracortical recordings. A durotomy was initiated using a 23G bent needle to pierce the dura. The dura was further removed using precision forceps and sharp micro scissors to expose S1. S1 was identified based on descriptions of Craner and Ray^47^ and Sauleau et al.^22^; the foundational model development is described elsewhere (W. Jensen, A. Hayward and C. R. Bjarkam, unpublished data). An electrode array with two shanks with eight channels each (E16-285-S2-L8-1100; ATLAS Neuroengineering) was lowered 3 mm into the brain using a manually driven micromanipulator. After 5 min to allow the brain tissue to adjust, the electrode was retracted so that the tip was at a depth of 1.5 mm. Electrophysiological recordings were conducted in anesthetized animals 40 min after electrode placement to allow the electrodes to settle in the tissue. The experimental timeline is shown in Supplementary Fig. 1.

### Electrophysiology

Bipolar electrical stimulation was applied with a 3 mA cathodic-first pulse of 100 µs duration and an anodic phase of 375 µA and 800 µs duration, separated by a 100 µs interpulse interval. Stimulation was repeated 200 times at 2 Hz, alternating between the ulnar and median nerve, with random interstimulus interval to avoid adaptation. Stimulation series were performed at 10 min intervals. A total of 3 stimulation series were recorded before intervention and 18 after. Cortical data were recorded through an RX5 Pentusa Base Station system (Tucker-Davis Technologies) at 25 kHz, then high pass filtered at 300 Hz. A manual threshold (threshold equal to three to five times the RMS value of the background noise level) was used for online spike detection.

### Histology

To determine in which layer the channels were located, histological analysis was performed for seven of the animals. A block around the electrode region of seven animals was cut and post-fixed in formalin (10% w/v) for 12 weeks. The tissue was then divided into 0.5 cm blocks, which were embedded in paraffin and sliced on a vibratome (10 µm thickness). Sections were Nissl stained using cresyl violet and slides were visualized by a slide scanner. NanoZoomer Digital Pathology View (version 2.2.1, Hamamatsu Photonics) was used for precise localization of the electrode contacts and identification of cortical layers^28^. Contact sites were marked by electrochemically damaging the tissue surrounding the contacts using prolonged 1 mA monophasic electrical stimulation.

### Data analysis

All spikes above the threshold were saved and used to construct PSTHs, using the spikes detected 50 ms before the peripheral stimulus and up to 450 ms after. All spikes detected after a single stimulus were divided into 1 ms bins and added together for each stimulation session consisting of 100 stimuli per nerve. The background activity was subtracted by removing the average spike count 50 ms to 5 ms before stimulus onset, to account for differences in thresholding.

Two peaks were visually distinguished in the PSTH, 10–20 ms after the stimulus and between 20 and 35 ms after the stimulus. PSTHs were, therefore, divided into two time windows: 10–19 ms and 21–35 ms after the stimulus. The peak amplitude was calculated as the maximum spike count within these time windows. The latency was calculated using the CoM for both time ranges, as follows:

$${\mathrm{CoM}}=\frac{1}{{\sum }_{t1}^{t2}{\mathrm{spikes}}}\mathop{\sum }\limits_{t1}^{t2}({\mathrm{spikes}}\times t),$$

where *t*1 and *t*2 are the lower and higher end of the time windows, ‘spikes’ is the spike count per bin and *t* is time.

Spikes recorded on all channels were averaged to investigate the effect of stimulation of the uninjured median and injured ulnar nerve on PSTH peak amplitude and latency. This is common practice for signals from the same brain area^14,26^. The data were divided into 30 min phases consisting of three stimulation sessions, one phase before and six phases after the intervention. PSTH of the three sessions in a phase were averaged, after which peak amplitude and CoM were extracted. For the analysis of the different layers, the responses to stimulation of the median and ulnar nerves were averaged, as there was no significant difference between them.

Channels that did not record a response to stimulation during any stimulation session throughout the experiment were removed. Eleven irresponsive channels were removed. Eight channels were from a single control experiment, where one shank was either misplaced or damaged. Two channels were located in layer I in a control animal and one channel was located in layer I in a NI experiment. To analyze whether responses differed per cortical layer, data from seven animals were used for which histology was performed. The number of channels per layer is presented in Table 1.

Spike sorting was performed in an automated manner using a custom-made analysis code. Principle component analysis was performed to identify the most relevant seven features: number of zero-crossings, peak width of the most prominent peak, amplitude and latency of the positive and negative peaks and whether the positive or negative polarity occurred first. The features that explained more than 10% of the variability were used in a *k*-means clustering algorithm. Few neurons per channel were expected, so the maximum number of clusters was set to five. The optimal number of clusters was identified using the silhouette method, which compares the similarity of the data within a cluster to the similarity between different clusters. Finally, the data were clustered into the optimal number of clusters identified by the silhouette method using *k*-means clustering.

### Statistical analysis

To determine whether there was a difference in excitability when stimulating the injured compared with the uninjured nerve, three-way repeated measures ANOVA was used. Dependent variables were CoM and peak amplitude. Between-subject factors were control and NI; within-subject factors were time (one measurement before and six after intervention) and (uninjured) median and (injured) ulnar nerve. The stimulation of the two different nerves was assumed to activate two distinct populations of neurons in S1. From the ten datasets collected, one dataset was excluded due to missing data, resulting in six intervention and three control datasets. Normality of the data was verified using the Shapiro–Wilk test and the Q–Q plots.

Since the brain response from different layers after the same stimulus violates the independence assumption, a mixed model was used for the statistical analysis of the seven datasets for which histological analysis was performed. Fixed factors in the mixed model were layer, intervention and time. Time was also a repeated effect, which was modeled using a first-order autoregressive covariance matrix to account for the dependence of the data. Both slope and intercept were added as a random factor, which improved the quality of the model. A backwards approach was used to find the appropriate model, where the least significant fixed factors were removed sequentially until only significant factors remained. The resulting models are provided in Table 2.

For all models with significant interactions and significant main effects, pairwise comparisons were performed. These comparisons were used to answer three questions for each of the dependent variables (peak amplitude and CoM of the early and late peak):

- Does the evoked response to electrical stimulation differ between cortical layers?
- Is there a difference in cortical responses between the NI intervention and control group within each cortical layer and phase?
- Does the response within each layer differ after the NI intervention compared with the baseline?

The statistical tests were performed using SPSS version 27. Multiple comparisons were adjusted using the Bonferroni correction. The differences were considered statistically significant when *P* < 0.05. The estimated marginal means and standard error of the mean are reported.

### Reporting summary

Further information on research design is available in the Nature Portfolio Reporting Summary linked to this article.

## Online content

Any methods, additional references, Nature Portfolio reporting summaries, source data, extended data, supplementary information, acknowledgements, peer review information; details of author contributions and competing interests; and statements of data and code availability are available at 10.1038/s41684-024-01440-0.

## Supplementary information

Supplementary Information Supplementary Fig. 1.

Reporting Summary

## Data Availability

The data that support the findings of this study are available from the corresponding author upon request.
