# Supplementary material for: Spared ulnar nerve injury results in increased layer III–VI excitability in the pig somatosensory cortex
Source: Lab Anim (NY). 2024 Sep 30;53(10):287–93. doi: 10.1038/s41684-024-01440-0 (PMC11442301; doi:10.1038/s41684-024-01440-0)

lab animal

Supplementary information

https://doi.org/10.1038/s41684-024-01440-0

Spared ulnar nerve injury results in increased layer III–VI excitability in the pig somatosensory cortex

In the format provided by the authors and unedited

Supplementary Fig. 1| Experimental timeline. The measurement blocks are colored blue and the surgical blocks are colored orange. Measurement blocks consist of one baseline recording and 6 post-SNI recordings up to 180 minutes after the intervention.

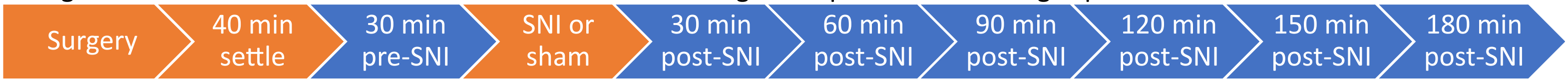

Supplement: Supplementary file 1 — Supplementary Fig. 1. [file 41684_2024_1440_MOESM1_ESM.pdf]
